# *Microorganisms*—A Forum for Understanding Microbial Life in All Its Forms

**DOI:** 10.3390/microorganisms1010001

**Published:** 2013-02-19

**Authors:** John Fuerst

**Affiliations:** *Founding Editor-in-Chief*, School of Chemistry and Molecular Biosciences, The University of Queensland, St. Lucia 4072, Queensland, Australia; E-Mail: j.fuerst@uq.edu.au

It is my great pleasure to welcome you to *Microorganisms*, a new open access journal, which is dedicated to microorganisms in all their forms and via any approach to their study.

Our present gene and genome-informed understanding of the Tree of Life has revealed the immense significance of cellular microorganisms, an insight initiated by the late Carl Woese, founder of the modern study of the evolutionary relationships of such microorganisms in their widest sense. This is especially so when we appreciate that of the three cellular Domains of life, Archaea, Bacteria and Eucarya, the first two of these are completely microbial. Even within the Eucarya, members of which are commonly referred to as eukaryotes, most phyla are predominantly unicellular or microscopically mycelial microorganisms, leaving only the rather fragile twigs of animals and plants at one extreme tip of the tree consisting of species living only a multicellular lifestyle mostly visible at macroscopic scale [1]. Even some metazoan animals, not the usual subject of microbiology, are microscopic and form a surprisingly significant part of the marine microscopic biosphere [2]. We are also beginning to appreciate more fully that viruses must be considered as quite complex microorganisms, perhaps having contributed to the evolution of the cellular life-forms more significantly than was thought [3]—even as a fourth and perhaps ancient domain of life [4]. So too, regardless of their controversial status as microorganisms, the mysterious prions must be considered for full understanding of the cellular and evolutionary biology of yeasts as well as mammalian cells [5], and even for understanding eukaryote epigenetics and its relations to evolutionary change [6].

A journal fully recognizing the broadly diverse yet connected nature of microorganisms in all their manifestations is more than ever needed for scientific progress, for the insights that only full appreciation of the breadth of the microbial spectrum can yield. Further, these microorganisms must be considered in both pure and applied aspects, whether giving benefits to us or causing disease, and concerning their place in nature via the wondrous tools of contemporary microbial ecology, as well as their experimental understanding via the laboratory and laboratory model organisms. Phenotypic properties including microbial physiology, proteomics, metabolomics and the insights of microbial cell biology and systems biology must be considered as well as genomics and phylogenomics. The biology and behaviour of microorganisms used in or relevant to biotechnology, food microbiology and industrial microbiology must also be encompassed. This inclusiveness may lead to insights into the evolutionary cell biology of the diverse phyla of life and their evolutionary relationships, answering some of the major questions posed for contemporary biology. *Microorganisms* aims to provide that broad and inclusive venue. 

The new online, open-access journal *Microorganisms* has been launched to provide an advanced publishing forum for original peer-reviewed articles from scientists involved in high-quality pure and applied research on any prokaryotic or eukaryotic microorganism or other non-cellular transmissible agent—bacterium, archaeon, fungus, protist, alga, virus or prion. It publishes reviews, research papers and communications. The scope of the journal encompasses any first-class research in microbial physiology, microbial ecology, microbial genetics, evolutionary microbiology, systems microbiology, medical microbiology, pharmaceutical microbiology and anti-microbial agents, industrial microbiology, agricultural microbiology, microbial biotechnology, food microbiology and environmental microbiology; and, of course, in evolutionary cell biology and evolutionary microbiology, the deep organizing principles of microbial biology. 
